# Predictors of health-related quality of life after burn injuries: a systematic review

**DOI:** 10.1186/s13054-018-2071-4

**Published:** 2018-06-14

**Authors:** Inge Spronk, Catherine M. Legemate, Jan Dokter, Nancy E. E. van Loey, Margriet E. van Baar, Suzanne Polinder

**Affiliations:** 1 Association of Dutch Burn Centres, Maasstad Hospital, Maasstadweg 21, 3079 DZ Rotterdam, the Netherlands; 2 Department of Public Health, Erasmus Medical Centre, ‘s-Gravendijkwal 230, 3015 CE Rotterdam, the Netherlands; 3 Department of Plastic, Reconstructive and Hand Surgery, Amsterdam Movement Sciences, VU University Medical Centre, De Boelelaan 1117, 1081 HV Amsterdam, the Netherlands; 4 Burn Centre, Maasstad Hospital, Maasstadweg 21, 3079 DZ Rotterdam, the Netherlands; 5 Association of Dutch Burn Centres, Red Cross Hospital, Vondellaan 13, 1942 LE Beverwijk, the Netherlands; 6 Department of Clinical Psychology, Utrecht University, Domplein 29, 3512 JE Utrecht, the Netherlands

**Keywords:** Burn injuries, Health-related quality of life, Predictors

## Abstract

**Background:**

Identifying predictors of health-related quality of life (HRQL) following burns is essential for optimization of rehabilitation for burn survivors. This study aimed to systematically review predictors of HRQL in burn patients.

**Methods:**

Medline, Embase, Web of Science, Cochrane, CINAHL, and Google Scholar were reviewed from inception to October 2016 for studies that investigated at least one predictor of HRQL after burns. The Quality in Prognostic Studies tool was used to assess risk of bias of included studies.

**Results:**

Thirty-two studies were included. Severity of burns, postburn depression, post-traumatic stress symptoms, avoidance coping, less emotional or social support, higher levels of neuroticism, and unemployment postburn were found to predict a poorer HRQL after burns in multivariable analyses. In addition, weaker predictors included female gender, pain, and a postburn substance use disorder. Risk of bias was generally low in outcome measurement and high in study attrition and study confounding.

**Conclusions:**

HRQL after burns is affected by the severity of burns and the psychological response to the trauma. Both constructs provide unique information and knowledge that are necessary for optimized rehabilitation. Therefore, both physical and psychological problems require attention months to years after the burn trauma.

**Electronic supplementary material:**

The online version of this article (10.1186/s13054-018-2071-4) contains supplementary material, which is available to authorized users.

## Background

Health-related quality of life (HRQL) is an important outcome measure of burns in both the short- and long-term [1, 2] and is increasingly studied. HRQL is a multidimensional concept that reflects an individual’s perception of how a disease affects his/her physical, psychological, and social well-being [3–5]. Insight into which factors determine HRQL after burns is useful for clinical practice, research, and policy making. Conceptual models have been developed in order to better understand HRQL and the variables that relate to HRQL in general [3, 6–8]. According to the revised Wilson and Clearly model for health-related quality of life, HRQL is influenced by individual and environmental characteristics, biological function, symptoms, functional status, and general health perceptions [3]. A recent study confirmed that this model is also applicable to burns [9].

Burns can have a considerable negative impact on daily activities and on both physical and psychosocial functioning [10–12]. HRQL domains are often impaired in the short-term. Most domains of HRQL improve in the longer-term, but also in the longer-term some aspects (e.g., physical and emotional role participation) have poor outcomes [13–15]. Burn injuries are thus associated with a significant physical and psychological burden.

The prediction of an individual’s ability to adjust to the consequences of their burn injury is important. Information regarding these predictors may help caregivers in selecting patients who require special attention in rehabilitation and in preparing patient-specific care plans [16]. Predictors of HRQL following burns have been examined in individual studies, but predictors of HRQL have not been systematically reviewed in the field of burns. Potential meaningful factors are the patient’s age and gender, percentage total body surface area (%TBSA) burned, length of hospital stay, body area affected, time since injury, and psychological impact of burns. However, it is not yet clear which predictive factors are most important [17–20]. Earlier recent reviews focused on the evolution and relevance of one specific HRQL instrument in burns [21], on HRQL outcomes in burns [19], and on HRQL instruments used and recovery patterns of HRQL in burns, without studying predictors. Therefore, the aim of the present study is to systematically review predictors of HRQL following burn injuries.

## Methods

This systematic review was conducted and is reported in line with the Preferred Reporting Items for Systematic Reviews and Meta-analyses (PRISMA) Statement [22] and has been registered on PROSPERO (ID CRD42016048065).

### Search strategy and inclusion criteria

The databases Medline, Embase, Web of Science, Cochrane, CINAHL, and Google Scholar were systematically searched using terms covering HRQL and burns (search strategy provided in Additional file 1) in October 2016. The search strategy was developed in collaboration with an experienced librarian. Original prognostic studies conducted in adult burn patients and focusing on at least one predictor of HRQL after burns were included. Studies had to be published in a peer-reviewed journal and written in English and were required to have used a generic or burn-specific instrument to assess HRQL. Outcomes had to be a regression or correlation coefficient of the relation of a predictor with HRQL. All kinds of predictors were considered.

### Selection of studies and data extraction

An experienced librarian performed the systematic search. After removal of duplicates, relevant articles were selected on the basis of title by one researcher (IS). Ten percent of the abstracts were independently evaluated by two researchers (IS and CL). Perfect agreement on inclusion was achieved (Cohen’s kappa coefficient = 1); therefore, one researcher evaluated the remaining abstracts (IS) [23]. In case of any doubt, a title or abstract was screened by a second researcher. Two researchers (IS and CL) independently performed screening of full text and extraction of data. The screening of these three steps was performed using the above mentioned inclusion criteria.

Data extraction included study characteristics (study type, country, sample size, assessment time points, length of follow-up), patient and burn characteristics (age, gender, hospital length of stay (LOS), %TBSA), details on HRQL instruments (type, number, general burn-specific HRQL, proxy), and predictors (number of predictors assessed, univariable and multivariable predictors, statistical methods). Discrepancies arising from decisions around inclusion or extraction of data were discussed with a third researcher (MvB) until resolved.

### Risk of bias

The Quality in Prognostic Studies (QUIPS) tool [24] was used to assess the risk of bias of the included studies. Two researchers (IS and CL) independently assessed the risk of bias of the six domains. The domains were rated as either low, moderate, or high risk of bias. A low risk was obtained when all items of a domain were scored as “low risk” [24]. A moderate risk was obtained when at least one up to a maximum of half of the items were rated as high or had an unknown risk of bias. A high risk was obtained when more than half of the items were rated as high or had an unknown risk of bias. Disagreements were resolved by discussion with a third researcher (MvB).

### Data analysis

First the characteristics and the risk of bias of all studies were tabulated. Then the predictor findings of studies using multivariable analysis were analyzed. Multivariable models were models that included at least two factors to predict HRQL. Predictors were divided into four categories: demographic, environmental, burn-specific, and psychological factors. If it was unclear whether associations were significant (*p* ≤ 0.05), results could not be included in our analysis. When more than one time point was used, the point closest to the most often used time points in other studies was chosen. Given the heterogeneity of predictors, HRQL instruments, and statistical reporting, meta-analyses could not be conducted. Therefore, a more qualitative approach was used: all predictors of each study were summarized on the basis of their direction and statistical significance [25, 26]. Predictors were scored having no statistically significant association (*p* > 0.05) with HRQL, a significant association (*p* ≤ 0.05) with a subscale of the HRQL instrument, or a significant association with the total HRQL instrument. Associations with the total HRQL instrument were weighted more heavily (Table 3). Due to the wide variety of predictors assessed among the included studies, only those predictors that were studied in more than one study were tabulated (Table 3). Predictors were considered strong when ≥ 67% of the associations were in the same direction and statistically significant and weak if ≥ 33 to < 67% of the associations fulfilled these conditions.

## Results

### Search results

**Fig. 1:**
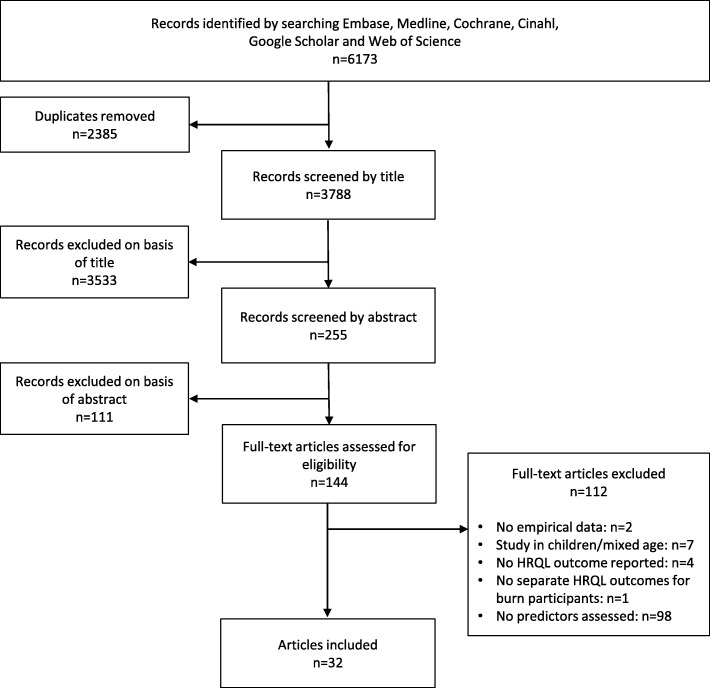
Flowchart showing the selection of studies

The initial database search netted 6173 records, including 3788 unique articles. Screening of titles and abstracts resulted in 144 potentially relevant articles (Fig. 1). Thirty-two of these were eligible after reading the full text. The main reason for exclusion was not studying predictors.

### Study characteristics

Sample sizes varied between 20 and 1051 patients, with most studies (75%) having a sample size below 200 patients (Table 1). In all except one study [27], more males than females were included. The mean %TBSA burned ranged from 8 to 84%. Eleven different HRQL instruments were used in the included studies. The most often used instruments were the Burn-specific Health Scale-Brief (BSHS-B; *n* = 17) and the Medical Outcome Study Short Form—36 items (SF-36; *n* = 11). Eighteen studies measured HRQL at one time point, whereas 13 measured HRQL two to six times. One study failed to describe their assessment point. The most used time points were at 3 months (*n* = 6), 6 months (*n* = 11), 12 months (*n* = 12), and 24 months (*n* = 7). Seventeen studies used an assessment point more than one year after the burn injury.

**Table 1:** Characteristics of included studies (*n* = 32)

Study	Country	Study population^a^	Mean %TBSA burned (SD)	HRQL instrument(s)^b^	Assessment time point(s)
Ahuja et al. 2016 [27]	India	*n* = 60 (M, 40%). Age, 18–65 years (median, 28 years)	Median, 30%	BSHS-RBA	Median, 10 months
Anzarut et al. 2005 [53]	Canada	*n* = 47 (M, 96%). Mean age 28 years	64% (2)	BSHS-A, SF-36	≥ 2 years after discharge
Blalock et al. 1994 [54]	USA	*n* = 254 (M, 74%). Mean age 39 years	19% (15)	BSHS	Mean, 8–9 months
Corry et al. 2010 [55]	USA	*n* = 171 (M, 70%). Age, 8–86 years (mean, 42 years)	15% (13); range, 1–74%	SF-36	Discharge, 1, 6, 12, and 24 months^c^
Cromes et al. 2002 [56]	USA	*n* = 110 (M, 84%). Mean age 38 years	24%	BSHS	2^d^, 6^d^, and 12^d^ months
Edgar et al. 2013 [17]	Australia	*n* = 1051, (M, 80%). Age, 15–89 years (mean, 37 years)	8% (11); range, 0–75%	BSHS-B, SF-36	1, 3, 6, 12, and 24 months^c^
Ekeblad et al. 2015 [29]	Sweden	*n* = 107 (M, 75%). Age, 19–89 years (mean 43 years)	23%; range 1–80%	BSHS-B, EQ-5D, SF-36	12 months
Finlay et al. 2014 [57]	Australia	*n* = 927 (M, 73%). Age, 16–83 years (mean, 32 years)	7% (10)	BSHS-B	Discharge, 1, 3^d^, 6, 12, and 24 months
Finlay et al. 2015 [58]	Australia	*n* = 224 (M, 83%). Age, 16–84 years (median 36 years)	Median, 4%; range 1–60%	BSHS-B	NA
Kildal et al. 2001 [59]	Sweden	*n* = 248 (M, 80%). Mean age 37 years	23% (16)	BSHS-B	Mean, 9.3 years (SD 4.8 years)
Kildal et al. 2004 [60]	Sweden	*n* = 166 (M, 80%). Mean age 50 years	25% (16)	BSHS-B	Mean, 11.4 years; range, 3 – 19 years
Kildal et al. 2005 [61]	Sweden	*n* = 161 (M, 79%). Age, 17–79 years (mean, 48 years)	24% (16); range, 1–85%	BSHS-B	Mean, 9.2 years; range 1–18 years
Knight et al. 2017 [62]	Australia	*n* = 41 (M, 81%). Age, 19–81 years (mean, 45 years)	8%	BSHS-B	12–24 months
Leblebici et al. 2006 [63]	Turkey	*n* = 22 (M, 64%). Mean age 25 years	28% (17)	SF-36	Mean, 21 months
Low et al. 2012 [64]	Sweden	*n* = 85 (M, 75%). Age, 19–89 years (mean, 45 years)	24% (20); range, 1–80%	BSHS-B	12 months
Moi et al. 2007 [65]	Norway	*n* = 95 (M, 82%). Mean age 44 years	19% (14)	BSHS-A	Mean, 47 months (SD 24 months)
Moi and Nilsen 2012 [9]	Norway	*n* = 95 (M, 82%). Mean age 44 years	19% (14)	BSHS-A, SF-36, QOLS	Mean, 47 months (SD 24 months)
Novelli et al. 2009 [66]	Italy	*n* = 30 (M, 60%). Mean age 42 years	32% (13)	SIP	Discharge, 3 months^d^
Orwelius et al. 2013 [28]	Sweden	*n* = 156 (M, 74%). Age, 16–90 years (mean, 46 years)	24% (19); range, 0–80%	SF-36	12^d^ and 24 months
Oster et al. 2011 [18]	Sweden	*n* = 89 (M, 77%). Mean age 43 years	25% (20)	EQ-5D	Admission, 3, 6, 12 months, and 2 to 7^d^ years
Oster et al. 2013 [30]	Sweden	*n* = 67 (M, 78%). Mean age 43 years	25% (20)	BSHS-B	6 and 12 months and 2 to 7^d^ years
Palmu et al. 2015 [67]	Finland	*n* = 92 (M, 70%). Mean age 46 years	10%	15D, EQ-5D, RAND-36	6 months
Renneberg et al. 2014 [68]	Germany	*n* = 265 (M, 72%). Age, 16–73 years (mean, 39 years)	14% (14); range, 1–76%	BSHS-B, SF-12	Admission, 6, 12, 24, and 36 months^c^
Ricci et al. 2014 [69]	Brazil	*n* = 73 (M, 69%). Mean age 38 years	14% (12)	BSHS-R	5 to 7 months
Roh et al. 2012 [70]	South Korea	*n* = 113 (M, 71%). Mean age 38 years	14% (12)	BSHS-B	1 month
Tahir et al. 2011 [71]	Pakistan	n = 99 (M, 68%). Age, 19–57 years (median, 30 years)	19%, range; 5–38%	SF-36	Admission, 5 and 6^d^ months
Van Loey et al. 2012 [20]	The Netherlands and Belgium	*n* = 244 (M, 73%). Mean age 39 years	12% (11); range 1–65%	EQ-5D	3 weeks, 3, 9, and 18 months^c^
Wasiak et al. 2014 [72]	Australia	*n* = 99 (M, 75%). Mean age 42 years	19%	BSHS-B, SF-36	Preburn and 12^d^ months
Willebrand et al. 2006 [73]	Sweden	*n* = 86 (M, 73%). Age, 15–85 years (mean, 43 years)	17% (14)	BSHS-B	Mean, 3.6 years (SD 1.2 years)
Willebrand and Ekselius 2011 [74]	Sweden	*n* = 94 (M, 76%). Age, 19–90 years (mean, 44 years)	23% (20)	BSHS-B, SF-36	6^d^, 12^d^, and 24^d^ months
Xie et al. 2012 [75]	China	*n* = 20 (M, 70%). Mean age 43 years	84% (10)	BSHS-B, SF-36	≥ 2 years after discharge
Zhang et al. 2014 [33]	China	*n* = 208 (M, 77%). Mean age 42 years	42% (27)	BSHS-B	≥ 2 years after discharge

^a^Study population: *n* sample size; *M* males; *NA* not applicable

^b^*15D* 15-dimensional health-related quality of life instrument, *BSHS* Burn-specific Health Scale, *BSHS-A* Burn-specific Health Scale—Abbreviated, *BSHS-B* Burn-specific Health Scale—Brief, *BSHS-RBA* Burn-specific Health Scale Revised, Brief and Adapted, *EQ-5D* EuroQol five dimensions questionnaire, *RAND-36* RAND 36-item health survey, *SIP* Sickness Impact Profile, *SF-10* Medical Outcome Study Short Form—10 items, *SF-12* Medical Outcome Study Short Form—12 items, *SF-36* Medical Outcome Study Short Form—36 items, *QOLS* Quality of Life Scale

^c^All measurement points were used as the dependent variable was long-term recovery pattern

^d^Measurement point used for predictor analysis in studies with ≥ 1 measurement point

### Risk of bias

The quality of included studies was in general moderate. In most studies risk of bias was moderate or high for the items “study attrition” and “study confounding” (Table 2). Positive aspects of the studies were the low risk of bias for the items “outcome measurement” and “statistical analysis and reporting”. None of the studies scored a low risk of bias on all items and one study had a low risk on all but one dimension [28].

**Table 2:** Risk of bias assessment according to the Quality of Prognostic Studies (QUIPS) tool (*n* = 32)

Study	Study Participation	Study attrition	Prognostic factor measurement	Outcome measurement	Study confounding	Statistical analysis and reporting	Total score
Ahuja et al. 2016 [27]	Low	Low	Moderate	Low	Moderate	Low	8
Anzarut et al. 2005 [53]	Moderate	Moderate	Moderate	Moderate	High	Moderate	13
Blalock et al. 1994 [54]	Moderate	High	Moderate	Low	High	Low	11
Corry et al. 2010 [55]	Moderate	High	Low	Low	Moderate	Low	10
Cromes et al. 2002 [56]	Moderate	High	Moderate	Low	High	Moderate	13
Edgar et al. 2013 [17]	Low	Low	Moderate	Low	Moderate	Low	8
Ekeblad et al. 2015 [29]	Low	Moderate	High	Low	High	Low	11
Finlay et al. 2014 [57]	Low	Moderate	Low	Low	Moderate	Low	8
Finlay et al. 2015 [58]	Low	Moderate	Low	Low	Moderate	Low	8
Kildal et al. 2001 [59]	Low	Moderate	Moderate	Low	Moderate	Low	9
Kildal et al. 2004 [60]	Low	Moderate	Moderate	Low	Moderate	Low	9
Kildal et al. 2005 [61]	Low	High	Moderate	Low	Moderate	Low	10
Knight et al. 2017 [62]	Moderate	High	Low	Low	Low	Low	9
Leblebici et al. 2006 [63]	Moderate	High	Moderate	Low	Low	Low	10
Low et al. 2012 [64]	Low	Moderate	Low	Low	Moderate	Low	8
Moi et al. 2007 [65]	Low	Moderate	Low	Low	Moderate	Low	8
Moi and Nilsen 2012 [9]	Low	Moderate	Moderate	Low	Moderate	Low	9
Novelli et al. 2009 [66]	High	High	Moderate	Low	High	Moderate	14
Orwelius et al. 2013 [28]	Low	Moderate	Low	Low	Low	Low	7
Oster et al. 2011 [18]	Low	Moderate	Moderate	Low	Moderate	Low	9
Oster et al. 2013 [30]	Low	Moderate	Moderate	Low	Moderate	Low	9
Palmu et al. 2015 [67]	Low	Moderate	Low	Low	Moderate	Moderate	9
Renneberg et al. 2014 [68]	Moderate	High	Low	Low	Low	Low	9
Ricci et al. 2014 [69]	Moderate	High	Moderate	Low	Moderate	Low	11
Roh et al. 2012 [70]	Moderate	High	Low	Low	Low	Low	9
Tahir et al. 2011 [71]	Low	High	Moderate	Low	High	Moderate	12
Van Loey et al. 2012 [20]	Low	High	Low	Low	Moderate	Low	9
Wasiak et al. 2014 [72]	Low	High	Low	Low	Moderate	Low	9
Willebrand et al. 2006 [73]	Low	High	Moderate	Low	Moderate	Low	10
Willebrand and Ekselius 2011 [74]	Low	Moderate	Moderate	Low	Moderate	Low	9
Xie et al. 2012 [75]	Moderate	Moderate	Low	Low	Low	Low	8
Zhang et al. 2014 [33]	Low	Moderate	Moderate	Low	Moderate	Moderate	10

The total score was composed of the sum of the domain scores, with low risk = 1, moderate risk = 2, and high risk = 3

### Predictors of HRQL

**Fig. 2:**
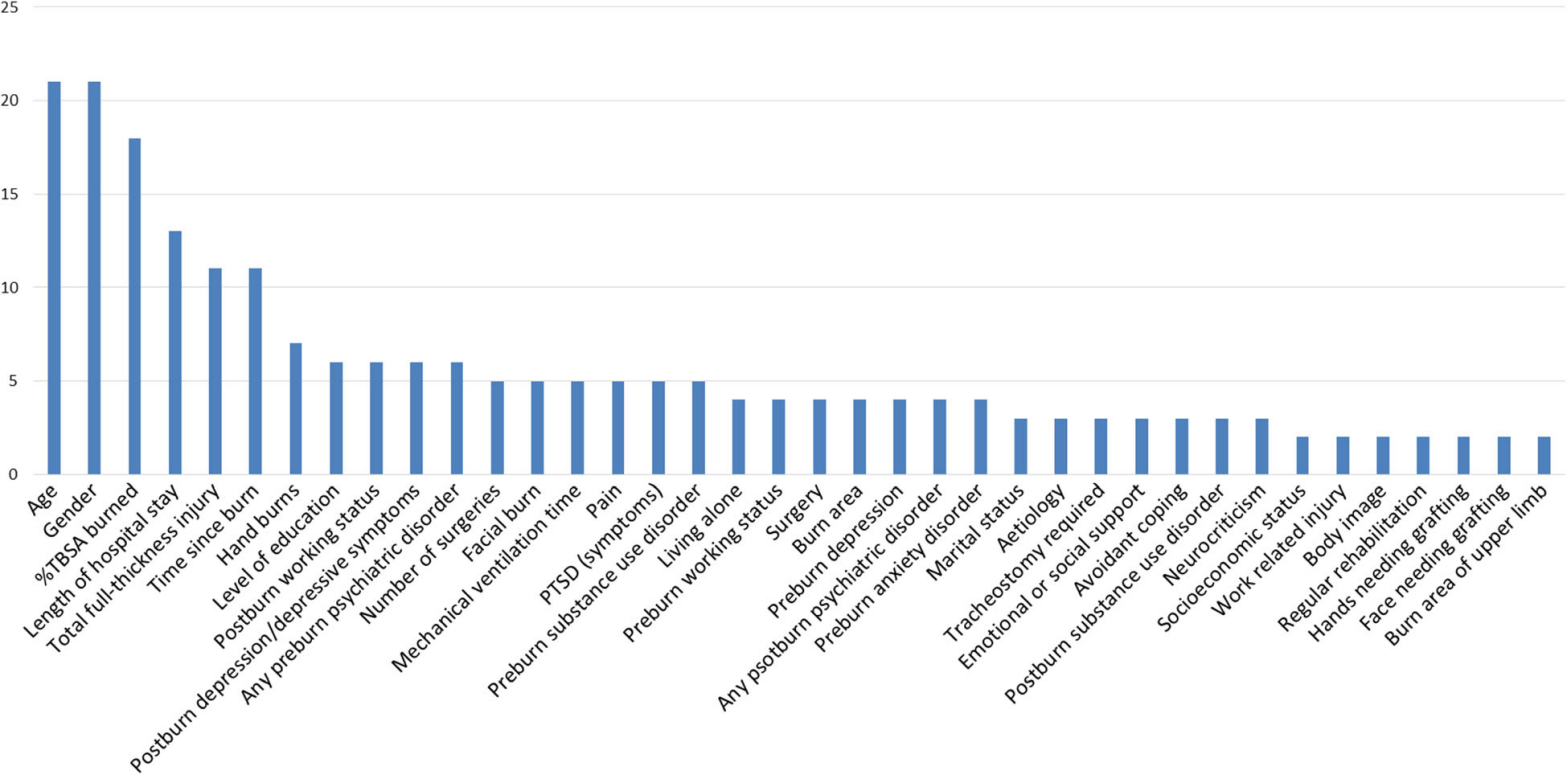
Predictors investigated in more than one multivariable predictive study

Twenty studies used multivariable analysis. One study [29] did not indicate significant (*p* ≤ 0.05) predictors and was therefore not included in our analyses. Three studies applied two different HRQL instruments, resulting in 22 different prediction studies. Eleven of these studies were based on four cohorts. Due to the low number of studies, all of these studies were included in the examination. The studies investigated between five and 42 predictors. Overall, 114 different predictors were investigated, of which 38 were studied in more than one study (Fig. 2). These were 16 burn-specific, 12 psychological, six demographic, and four environmental factors (Table 3, Additional file 2).

**Table 3:** Summary of 19 multivariable predictive studies of HRQL in adult burn patients

QUIPS score	8	8	8	8	8	9	9	9	9	9	9	9	9	9	9	9	10	10	10	12	13	13
	Edgar 2013 (SF-36)^1^	Edgar 2013 (BSHS-B)^1^	Finlay 2015 (BSHS-B)^1^	Low 2012 (BSHS-B)^4^	Xie 2012 (SF-36)	Renneberg 2014 (SF-12)	Van Loey 2012 (EQ-5D)	Kildal 2004 (BSHS-B)^2^	Wasiak 2014 (SF-36)^3^	Wasiak 2014 (BSHS-B)^3^	Moi 2012(QOLS)	Palmu 2015(RAND-36)	Oster 2011(EQ-5D index)^4^	Oster 2011(EQ-5D VAS)^4^	Oster 2013(BSHS-B)^4^	Knight 2016(BSHS-B)	Zhang 2014(BSHS-B)	Kildal 2005(BSHS-B)^2^	Willebrand 2006(BSHS-B)	Tahir 2011(SF-36)	Cromes 2002(BSHS)	Anzarut 2005(SF-36)
Demographic factors																						
Increasing age	–	+	0	+/−	+	0	--	?	--	--	0	?	0	0	0	0	0	?	–	0		–
Male gender	+	++	++	+	0	+	++	?	+	++	0	?	0	0	0	++	++	?	+	0		0
Married					0							0					0					
Living alone											0		0	0	0							
Low level of education					0							0	0	0	0		0					
Rehabilitation					0												0					
Environmental factors																						
Low socioeconomic status					0												0					
Work related injury																	0					0
Preburn working status												0	0	++	0							
Working status postburn					+						0		++	++	+		0					
Burn-specific factors																						
High %TBSA burned	--	--	0	–	0		0		0	0	0	?	0	0	0	0	--		–	0		0
Full-thickness injury			0	–	0				--	0	0		0	0	0		0					–
Longer length of hospital stay				–	+				0	0	0	0	--	0	--	--	0			--		0
Surgery	+	++	--														0					
Number of surgeries							--		0	0	0									--		
Burn area								?				0						?		--		
Hand burns					0							–	0	0	+		--					0
Hands needing grafting					0																	0
Facial burns					0								0	0			0					0
Face needing grafting					+																	0
Upper limb burn			++														0					
Mechanical ventilation					0				0	0							--					0
Tracheostomy required					0						0											0
Pain											0		--	0			--				0	
Etiology					0							0								0		
Longer time since burn	0	++			0			?					0	0	0		0	?	0			0
Psychological factors																						
Any preburn psychiatric disorder				0								0	0	0	0	--						
Any postburn psychiatric disorder				0								0	0	0								
Post-traumatic stress disorder or symptoms							--					?	0	--	–							
Preburn depression				–								?	0	0								
Postburn depression or depressive symptoms						–	--					--	0	0	–							
Preburn substance use disorder				–								?	0	0	0							
Postburn substance use disorder												?	0	--								
Preburn anxiety disorder				–									0	0	0							
Avoidant coping						–												–	–			
Emotional or social support																	0	+				+
Neuroticism						–		--											–			
Body image											0											0

Studies are ordered according to the QUIPS score and in addition to the number of patients included. Psychological disorders and symptom levels of depression and post-traumatic stress disorder were taken together. Avoidance coping includes post-traumatic stress disorder-avoidance, avoidance, and fear avoidance

*++* positive statistically significant correlation(*p* ≤ 0.05) with HRQL, *+* positive statistically significant correlation(*p* ≤ 0.05) with a domain(s) of HRQL only, *0* no statistically significant correlation(*p* > 0.05) with HRQL, *--* negative statistically significant correlation(*p* ≤ 0.05) with HRQL, *−* negative statistically significant correlation(*p* ≤ 0.05) with a domain(s) of HRQL only, *?* direction of correlation not reported, *%TBSA* percentage total body surface area

^1^Based on the same dataset, ^2^based on the same dataset, ^3^based on the same dataset, ^4^based on the same dataset

#### Demographic factors

The most studied demographic factors were age (*n* = 21) and gender (*n* = 21). The studies were inconsistent on whether age is a predictor for HRQL. Among the studies that studied gender, 11 found that male gender was associated with a better HRQL and three reported an association but failed to describe the direction. Marital status, living alone, rehabilitation, and level of education had no significant association with HRQL.

#### Environmental factors

The only environmental factor that showed an association with HRQL was postburn working status [18, 30]. Four studies reported that having a job postburn was associated with a better HRQL, and two did not find an association. Preburn working status was only found to relate to a better HRQL in one of the four studies examining this predictor and none of the studies found a relation between socioeconomic status or work-related injury and HRQL.

#### Burn-specific factors

The %TBSA burned is the most often studied burn-specific predictor (*n* = 18). Twelve studies found no association with HRQL, whereas five found a lower HRQL in more severely burned patients and one failed to describe the direction of the association. Somewhat more evidence exists on the LOS. Seven out of the 13 studies reported a lower HRQL after a longer LOS. Both surgery and number of surgeries were studied as predictors. Two studies reported a positive association between surgery and HRQL, whereas one study reported a negative association and one did not find an association. A higher number of surgeries resulted in a decreased HRQL in two studies. Three other studies, however, found no statistically significant association. Five individual predictors (LOS, %TBSA burned, full-thickness injury, surgery, and number of surgeries) are all indicators of burn severity. The cluster burn severity is a significant predictor of a diminished HRQL in 13 out of the 18 studies that investigated this predictor. Having pain as a predictor was investigated in five studies. Two found that patients that reported pain had a lower HRQL and three did not find an association. Evidence on other burn factors, including full-thickness injury, time since burn, hand burns, face needing grafting, upper limb burns, and mechanical ventilation was inconsistent. Studies found no association between either etiology, hands needing grafting, facial burns, or tracheostomy required and HRQL.

#### Psychological factors

Postburn depression or depressive symptoms and any preburn psychiatric disorder were the most often studied psychological factors (*n* = 6). Four out of the six studies that investigated postburn depression reported an association with impaired HRQL. Evidence also exists for higher levels of neuroticism and avoidance coping as predictors. The three studies that investigated these predictors all reported associations with poorer HRQL. Posttraumatic stress symptoms and less emotional or social support were also associated with diminished HRQL in the majority of studies. There was less evidence on preburn psychological factors (any psychiatric disorder, depression, substance use disorder, and anxiety disorder) and HRQL. Studies were inconsistent on postburn substance use disorder as a predictor and no association was found between any postburn psychiatric disorder and HRQL.

## Discussion

This study aimed to systematically review predictors of HRQL following burn injuries. Thirty-two studies were included and 114 predictors were investigated in 19 studies using multivariable analysis. Among burn patients, burn severity and psychological factors and, to a lesser extent, demographic and environmental factors are related to HRQL. Severity of burns, postburn depression, posttraumatic stress symptoms, avoidance coping, less emotional or social support, higher levels of neuroticism, and unemployment postburn were found to predict poorer HRQL after burns. In addition, some weaker predictors, including female gender, pain, and a postburn substance use disorder, were identified. Other demographic and environmental factors showed in general no significant association with HRQL and the evidence was inconclusive on other burn-specific and psychological factors. The quality of these studies was in general moderate.

This review clearly indicates that the severity of burns is a strong predictor of HRQL following burns. More severe burns generally result in a poorer HRQL. It is not yet clear, however, which individual severity predictor (e.g., LOS, %TBSA burned, number of surgeries) is best to indicate the severity of burns. By studying the multivariable results, the most optimal predictor becomes visible. The optimal predictor differed among the studies. The most consistent severity indicators for the prediction of HRQL seems to be LOS and number of surgeries. In the general trauma population, LOS is also a predictor of HRQL [31, 32] and there are some indications that surgical procedures predict a diminished HRQL [32]. The evidence regarding burn size was inconclusive; %TBSA burned was found to be negatively associated with HRQL in a minority (29%) of the studies. The other studies did not report a statistical significant association. It is remarkable that three out of the five larger studies (> 200 patients) reported a negative association, suggesting that %TBSA burned is a predictor of diminished HRQL after burns. However, it is questionable whether %TBSA burned is a good proxy for the severity of burns. It reflects the sum of the estimated percentage of full and partial thickness burns; it does not distinguish between deep and superficial wounds. Other burn-specific factors, including LOS or number of surgeries, may be better predictors [20]. Or possibly a combination of severity indicators may be the best predictor. There are also indications that having pain is a predictor for having a poorer HRQL after burns [18, 33]. It is known from other fields that patients who have severe continuing pain often also have a low HRQL [34, 35]. Other burn-specific factors, including body region burned, etiology, and longer time since burn did not generally seem to influence HRQL to a large extend.

Psychological factors are also important predictors for HRQL following burns. Five of the seven strong predictors are psychological factors, including postburn depression, posttraumatic stress symptoms, avoidance coping, less emotional or social support, and higher levels of neuroticism. These psychological factors are also predictors in other trauma populations [36–39]. Also, a postburn substance use disorder seems to be a predictor of an impaired HRQL, although evidence regarding this factor is weaker, both for burns and for trauma in general [40]. The often traumatic nature of burns may result in induced psychopathological responses [41], which is related to a poorer HRQL. Psychological burden can be caused by pain, grief, change of body image, self-blame, feelings of guilt, social isolation during hospital admission, or permanent physical disabilities [41]. In addition, earlier studies showed an association between psychological and physical burden. Psychological burden was associated with delayed wound healing [42], with greater physical impairment and role disruption [43], with slower physical recovery [43], and with poorer postburn adjustment [44]. The underlying reasons for this association is not yet clear. On the one hand, psychological distress might be influenced by physical problems [45]; those who appraise their injuries as more severe might have an increased risk of psychological problems. On the other hand, individuals with psychological problems might appraise their condition as worse and their recovery as less complete, and might have a decreased intention to be involved in rehabilitation [43]. Regardless of the underlying reasons for this relationship, increased psychological burden may result in an impaired HRQL.

The only demographic predictor of HRQL after burns was gender. Females reported a poorer HRQL after burns compared to males. This finding was also found in a recent study focusing on gender differences in HRQL outcomes in burn patients [46]. Reasons for females experiencing an impaired HRQL after burns are not clear. An explanation might be that females’ willingness to report problems is greater [47] or that women find it harder to live with a mutilated body. Females also reported higher levels of fatigue and higher mortality rates after burn injuries [47, 48]. Besides, poorer outcomes in females have been shown in injury studies in general [49, 50]. No strong conclusion could be drawn on the impact of age on HRQL after burns. Some studies reported better HRQL in younger adults, whereas others reported no or an adverse relationship. These inconsistent results are also seen in the general trauma population [31, 38, 40, 51].

Theoretically you would expect burn-specific instruments to be more sensitive to the consequences of burns. Thus, more statistically significant associations with HRQL measured by a burn-specific instrument would be expected. This was seen in the present study. Burn-specific instruments had a higher proportion of significant associations in multivariable studies. Forty-nine (47%) significant associations out of the 104 studied associations were found when HRQL was measured with a burn-specific instrument. For generic instruments, 45 (28%) out of the 163 studied associations were significant. The burn-specific instruments thus seem to be more sensitive compared to the generic instruments used. This finding is in line with the results of an earlier study that compared the BSHS-B against the SF-36 [52]. That study showed that SF-36 summary scores were less sensitive than the BSHS-B total score. The domain scores of the SF-36, however, were more sensitive than the domain scores of the BSHS-B [52]. Most included studies in the present review used SF-36 summary scores and BSHS-B domain scores.

The risk of bias of included studies was generally moderate. It was remarkable that none of the studies had an overall low risk of bias. In general, the risk of bias was moderate. A moderate or high risk of bias was seen particularly in the domains “study attrition” and “study confounding”. Only a minority of the studies set hypotheses before testing predictors and only a few underpinned their search for predictors with the available literature. Most studies did not report how missing data were handled. Besides, confounders were often not defined, attempts to collect information on patients who dropped out were not described, and key characteristics on those lost to follow-up were not reported. Future studies should include these factors in order to decrease the risk of bias and improve the overall study quality. Another issue was the use of multivariable analysis in 20 of the 32 included studies, indicating that 38% only used univariable analysis. As HRQL is a multifactorial concept, it is likely that HRQL is influenced by several factors and therefore multivariable analysis seems indicated. Univariable analysis is not very informative due to relations among the predictors.

### Strengths and limitations

A strength of this study is that it presents a comprehensive overview of predictors of a HRQL following burn injuries. Relevant literature databases were searched by an experienced librarian and quality was assessed using the widely applied QUIPS tool. A limitation is the exclusion of studies written in languages other than English, which might have resulted in missed studies published in other languages. Another limitation is the absence of a formal meta-analysis. Due to variation in instruments, time points, and data presentation in combination with the low number of studies, it was not possible to formally pool the results using meta-analysis. The examination of predictors on the basis of their direction and statistical significance that we applied does not take into account the sample size of the study nor the strength of predictors. However, we have checked that our main outcomes were not conditioned on sample size, risk of bias, or studies in the same dataset (Table 3). Due to the wide variation of assessment time points and the limited availability of short-term predictive studies, we were unable to study whether predictors differ in the short- and long-term.

## Conclusions

HRQL after burn injuries is particularly affected by the severity of burns and the psychological response of an individual to the trauma. Both constructs provide unique information and knowledge that is necessary for optimized follow-up treatment and rehabilitation. Therefore, a comprehensive approach, including both physical and psychological care, is indicated in the aftermath of burns. Screening of patients during follow-up is valuable to identify those patients who are in need of extra rehabilitation care. Patient-oriented treatment should be given and information on HRQL should be used to enhance patient-centered decision making.

To gain further insight in individual predictors and how they are correlated with each other, future studies should be based on the best available literature or on a theoretical framework, use larger sample sizes, and ensure high methodological quality. As it is hard to collect large samples in burns, combining several existing datasets is highly recommended.

## Additional files

Additional file 1: Search strategy. (DOCX 24 kb)

Additional file 2: Summary of 19 multivariable predictive studies of HRQL in adult burn patients according to time assessment points. (DOCX 43 kb)

## Abbreviations

%TBSA: Percentage of total body surface area
BSHS-B: Burn-specific Health Scale-Brief
HRQL: Health-related quality of life
LOS: Hospital length of stay
SF-36: Medical Outcome Study Short Form—36 items
QUIPS: Quality in Prognostic Studies
