# Supplementary material for: Predictors of health-related quality of life after burn injuries: a systematic review
Source: Crit Care. 2018 Jun 14;22:160. doi: 10.1186/s13054-018-2071-4 (PMC6000969; doi:10.1186/s13054-018-2071-4)
Supplement: Supplementary file 2 — Summary of 19 multivariable predictive studies of HRQL in adult burn patients according to time assessment points. (DOCX 43 kb) [file 13054_2018_2071_MOESM2_ESM.docx]

**Additional file 2. Summary of 19 multivariable predictive studies of HRQL in adults burn patients according to time assessment points**

| **Time point (months)** | 6 | 6 | 12 | 12 | 12 | 12 | 12-24 |  | >24 | >24 | >24 | 43 | 47 | 24-84 | 24-84 | 24-84 | 110 | 137 |  | Rec | Rec | Rec | Rec | NA |
| --- | --- | --- | --- | --- | --- | --- | --- | --- | --- | --- | --- | --- | --- | --- | --- | --- | --- | --- | --- | --- | --- | --- | --- | --- |
|  | Palmu 2015 (RAND-36) | Tahir 2011 (SF-36) | Cromes 2002 (BSHS) | Wasiak 2014 (SF-36)^3^ | Wasiak 2014 (BSHS-B)^3^ | Low 2012 (BSHS-B)^4^ | Knight 2016 (BSHS-B) |  | Zhang 2014 (BSHS-B) | Anzarut 2005 (SF-36) | Xie 2012 (SF-36) | Willebrand 2006 (BSHS-B) | Moi 2012 (QOLS) | Oster 2011 (EQ-5D index)^4^ | Oster 2011 (EQ-5D VAS)^4^ | Oster 2013 (BSHS-B)^4^ | Kildal 2005 (BSHS-B)^2^ | Kildal 2004 (BSHS-B)^2^ |  | Edgar 2013 (SF-36)^1^ | Edgar 2013 (BSHS-B)^1^ | Renneberg 2014 (SF-12) | Van Loey 2012 (EQ-5D) | Finlay 2015 (BSHS-B)^1^ |
| **Demographic factors** |  |  |  |  |  |  |  |  |  |  |  |  |  |  |  |  |  |  |  |  |  |  |  |  |
| Increasing age | ? | 0 |  | -- | -- | +/- | 0 |  | 0 | - | + | - | 0 | 0 | 0 | 0 | ? | ? |  | - | + | 0 | -- | 0 |
| Male gender | ? | 0 |  | + | ++ | + | ++ |  | ++ | 0 | 0 | + | 0 | 0 | 0 | 0 | ? | ? |  | + | ++ | + | ++ | ++ |
| Married | 0 |  |  |  |  |  |  |  | 0 |  | 0 |  |  |  |  |  |  |  |  |  |  |  |  |  |
| Living alone |  |  |  |  |  |  |  |  |  |  |  |  | 0 | 0 | 0 | 0 |  |  |  |  |  |  |  |  |
| Low level of education | 0 |  |  |  |  |  |  |  | 0 |  | 0 |  |  | 0 | 0 | 0 |  |  |  |  |  |  |  |  |
| Rehabilitation |  |  |  |  |  |  |  |  | 0 |  | 0 |  |  |  |  |  |  |  |  |  |  |  |  |  |
|  |  |  |  |  |  |  |  |  |  |  |  |  |  |  |  |  |  |  |  |  |  |  |  |  |
| **Environmental factors** |  |  |  |  |  |  |  |  |  |  |  |  |  |  |  |  |  |  |  |  |  |  |  |  |
| Low socioeconomic status |  |  |  |  |  |  |  |  | 0 |  | 0 |  |  |  |  |  |  |  |  |  |  |  |  |  |
| Work related injury |  |  |  |  |  |  |  |  | 0 | 0 |  |  |  |  |  |  |  |  |  |  |  |  |  |  |
| Preburn working status | 0 |  |  |  |  |  |  |  |  |  |  |  |  | 0 | ++ | 0 |  |  |  |  |  |  |  |  |
| Working status postburn |  |  |  |  |  |  |  |  | 0 |  | + |  | 0 | ++ | ++ | + |  |  |  |  |  |  |  |  |
|  |  |  |  |  |  |  |  |  |  |  |  |  |  |  |  |  |  |  |  |  |  |  |  |  |
| **Burn-specific factors** |  |  |  |  |  |  |  |  |  |  |  |  |  |  |  |  |  |  |  |  |  |  |  |  |
| High %TBSA burned | ? | 0 |  | 0 | 0 | - | 0 |  | -- | 0 | 0 | - | 0 | 0 | 0 | 0 |  |  |  | -- | -- |  | 0 | 0 |
| Full-thickness injury |  |  |  | -- | 0 | - |  |  | 0 | - | 0 |  | 0 | 0 | 0 | 0 |  |  |  |  |  |  |  | 0 |
| Longer length of hospital stay | 0 | -- |  | 0 | 0 | - | -- |  | 0 | 0 | + |  | 0 | -- | 0 | -- |  |  |  |  |  |  |  |  |
| Surgery |  |  |  |  |  |  |  |  | 0 |  |  |  |  |  |  |  |  |  |  | + | ++ |  |  | -- |
| Number of surgeries |  | -- |  | 0 | 0 |  |  |  |  |  |  |  | 0 |  |  |  |  |  |  |  |  |  | -- |  |
|  |  |  |  |  |  |  |  |  |  |  |  |  |  |  |  |  |  |  |  |  |  |  |  |  |
| Burn area | 0 | -- |  |  |  |  |  |  |  |  |  |  |  |  |  |  | ? | ? |  |  |  |  |  |  |
| Hand burns | - |  |  |  |  |  |  |  | -- | 0 | 0 |  |  | 0 | 0 | + |  |  |  |  |  |  |  |  |
| Hands needing grafting |  |  |  |  |  |  |  |  |  | 0 | 0 |  |  |  |  |  |  |  |  |  |  |  |  |  |
| Facial burns |  |  |  |  |  |  |  |  | 0 | 0 | 0 |  |  | 0 | 0 |  |  |  |  |  |  |  |  |  |
| Face needing grafting |  |  |  |  |  |  |  |  |  | 0 | + |  |  |  |  |  |  |  |  |  |  |  |  |  |
| Upper limb burn |  |  |  |  |  |  |  |  | 0 |  |  |  |  |  |  |  |  |  |  |  |  |  |  | ++ |
|  |  |  |  |  |  |  |  |  |  |  |  |  |  |  |  |  |  |  |  |  |  |  |  |  |
| Mechanical ventilation |  |  |  | 0 | 0 |  |  |  | -- | 0 | 0 |  |  |  |  |  |  |  |  |  |  |  |  |  |
| Tracheostomy required |  |  |  |  |  |  |  |  |  | 0 | 0 |  | 0 |  |  |  |  |  |  |  |  |  |  |  |
| Pain |  |  | 0 |  |  |  |  |  | -- |  |  |  | 0 | -- | 0 |  |  |  |  |  |  |  |  |  |
| Aetiology | 0 | 0 |  |  |  |  |  |  |  |  | 0 |  |  |  |  |  |  |  |  |  |  |  |  |  |
| Longer time since burn |  |  |  |  |  |  |  |  | 0 | 0 | 0 | 0 |  | 0 | 0 | 0 | ? | ? |  | 0 | ++ |  |  |  |
|  |  |  |  |  |  |  |  |  |  |  |  |  |  |  |  |  |  |  |  |  |  |  |  |  |
| **Psychological factors** |  |  |  |  |  |  |  |  |  |  |  |  |  |  |  |  |  |  |  |  |  |  |  |  |
| Any preburn psychiatric disorder | 0 |  |  |  |  | 0 | -- |  |  |  |  |  |  | 0 | 0 | 0 |  |  |  |  |  |  |  |  |
| Any postburn psychiatric disorder | 0 |  |  |  |  | 0 |  |  |  |  |  |  |  | 0 | 0 |  |  |  |  |  |  |  |  |  |
| Post-traumatic stress disorder or symptoms | ? |  |  |  |  |  |  |  |  |  |  |  |  | 0 | -- | - |  |  |  |  |  |  | -- |  |
| Preburn depression | ? |  |  |  |  | - |  |  |  |  |  |  |  | 0 | 0 |  |  |  |  |  |  |  |  |  |
| Postburn depression or depressive symptoms | -- |  |  |  |  |  |  |  |  |  |  |  |  | 0 | 0 | - |  |  |  |  |  | - | -- |  |
| Preburn substance use disorder | ? |  |  |  |  | - |  |  |  |  |  |  |  | 0 | 0 | 0 |  |  |  |  |  |  |  |  |
| Postburn substance use disorder | ? |  |  |  |  |  |  |  |  |  |  |  |  | 0 | -- |  |  |  |  |  |  |  |  |  |
| Preburn anxiety disorder |  |  |  |  |  | - |  |  |  |  |  |  |  | 0 | 0 | 0 |  |  |  |  |  |  |  |  |
|  |  |  |  |  |  |  |  |  |  |  |  |  |  |  |  |  |  |  |  |  |  |  |  |  |
| Avoidant coping |  |  |  |  |  |  |  |  |  |  |  | - |  |  |  |  | - |  |  |  |  | - |  |  |
| Emotional or social support |  |  |  |  |  |  |  |  | 0 | + |  |  |  |  |  |  | + |  |  |  |  |  |  |  |
| Neuroticism |  |  |  |  |  |  |  |  |  |  |  | - |  |  |  |  |  | -- |  |  |  | - |  |  |
| Body image |  |  |  |  |  |  |  |  |  | 0 |  |  | 0 |  |  |  |  |  |  |  |  |  |  |  |

*Note.* Studies are ordered according to the QUIPS score and in addition to the number of patients included. Psychological disorders and symptom levels of depression and post-traumatic stress disorder were taken together. Avoidant coping includes post-traumatic stress disorder-avoidance, avoidance and fear avoidance.

++ positive statistically significant correlation (p ≤ 0.05) with HRQL, + positive statistically significant correlation (p ≤ 0.05) with a domain(s) of HRQL only, 0 no statistically significant correlation (p > 0.05) with HRQL,-- negative statistically significant correlation (p ≤ 0.05) with HRQL, - negative statistically significant correlation (p ≤ 0.05) with a domain(s) of HRQL only, ? direction of correlation not reported, %TBSA = percentage total body surface area

^1^Based on the same dataset, ^2^based on the same dataset, ^3^based on the same dataset, ^4^based on the same dataset.
